# Applicability of time-restricted eating for the prevention of lifestyle-dependent diseases in a working population: results of a pilot study in a pre-post design

**DOI:** 10.3205/000291

**Published:** 2021-03-29

**Authors:** Dorothea Kesztyüs, Eva Vorwieger, Dorothée Schönsteiner, Markus Gulich, Tibor Kesztyüs

**Affiliations:** 1 Ulm University Medical Center, Institute of General Practice, Ulm, Germany; 2 Ulm University Medical Center, Clinical Chemistry, Ulm, Germany; 3 Georg August University Göttingen, Medical Center, Institute of Medical Informatics, Göttingen, Germany

**Keywords:** time-restricted eating, fasting, pilot project, feasibility study, occupational groups, adults, treatment adherence and compliance, health-related quality of life

## Abstract

**Background:** The ongoing epidemic of lifestyle-dependent diseases in industrialized countries threatens to overtax the health and social systems of these nations. New approaches beyond the usual therapeutic and preventive measures which have been applied so far must be tested. A paradigm shift with regard to nutrition and associated illness is overdue. Time-restricted eating (TRE) offers a low-threshold and easy-to-implement lifestyle change which may have what it takes for broad, population-wide applicability and a widely diversified range of possible effects. In this pilot study, we examine the feasibility and adherence of TRE in healthy adult employees.

**Methods:** Pre-post design study with healthy volunteers from the staff of Ulm University and Ulm University Hospital. Participants were asked to reduce their daily eating time to 8–9 hours for three months. Surrounding the eating time, they were allowed drinks other than water for 12 hours, and water for the rest of the day. Anthropometric measurements were taken by trained staff, and blood samples were taken at baseline and follow-up. Pre- and post-data on lifestyle, health and health-related quality of life (HRQoL, recorded with the Visual Analog Scale (VAS) of the EuroQol 5-Dimension (EQ-5D)), and attitudes towards TRE were collected in questionnaires. During the course of the study, timing of the first and the last meal, as well as sleep duration and quality, were assessed in diaries. Primary outcome was the percentage of days with achievement of the fasting goal out of the total number of days recorded per participant.

**Results:** Sixty-three participants (aged 47.8±10.5 years, 86% female) were recruited and started the intervention immediately after the baseline assessment. Two persons dropped out while all others finished the study. Ratings of compatibility of TRE with professional activities were good in 78% of participants, neither good nor difficult in 3%, and 18% reported to have encountered some difficulties. On average, the fasting target was reached on 72.2±18.9% of the recorded days. After three months of TRE, participants showed moderate reductions in weight (–1.3±2.3 kg, p≤0.001) and waist circumference (–1.7±3.2 cm, p≤0.001). HRQoL increased significantly by 5.8±12.4 (p=0.008) points between baseline and follow-up.

**Conclusion:** TRE is feasible and well accepted, even in regularly employed persons, and may improve HRQoL. TRE may help to reduce obesity and abdominal obesity in adult working people, thereby preventing lifestyle-dependent diseases; however, volunteers need more guidance to increase effects.

## Zusammenfassung

**Hintergrund:** Die anhaltende Epidemie lebensstilabhängiger Krankheiten in den Industrieländern droht, die Gesundheits- und Sozialsysteme dieser Nationen zu überfordern. Neue Ansätze, die über die bisher üblichen therapeutischen und präventiven Maßnahmen hinausgehen, müssen erprobt werden. Ein Paradigmenwechsel in Bezug auf Ernährung und damit verbundene Krankheiten ist überfällig. Zeitlich begrenzte Nahrungsaufnahme (TRE) bietet eine niedrigschwellige und leicht umsetzbare Lebensstiländerung, die die Möglichkeit zu einer breiten, bevölkerungsweiten Anwendbarkeit und einem breit gefächerten Spektrum positiver Auswirkungen haben kann. In dieser Pilotstudie untersuchen wir die Durchführbarkeit und Adhärenz von TRE bei gesunden erwachsenen Arbeitnehmerinnen und Arbeitnehmern.

**Methoden:** Studie im Prä-Post-Design mit gesunden Freiwilligen des Personals der Universität Ulm und des Universitätsklinikums Ulm. Teilnehmerinnen und Teilnehmer wurden gebeten, den Zeitraum ihrer täglichen Nahrungsaufnahme für drei Monate auf 8–9 Stunden zu begrenzen. Um die Essensphase herum durften sie 12 Stunden lang andere Getränke als Wasser trinken und für den Rest des Tages Wasser. Anthropometrische Messungen wurden von geschultem Personal durchgeführt und Blutproben wurden bei der Eingangs- und Nachuntersuchung entnommen. Prä- und Post-Daten zu Lebensstil, Gesundheit und gesundheitsbezogener Lebensqualität (HRQoL, erfasst mit der Visuellen Analogskala (VAS) des EuroQol 5-Dimension (EQ-5D)), sowie Einstellungen zu TRE wurden in Fragebögen erhoben. Der Zeitpunkt der ersten und letzten Mahlzeit sowie die Schlafdauer und -qualität wurden in Tagebüchern erfasst. Primärer Endpunkt war der prozentuale Anteil der Tage mit Erreichen des Fastenziels an der Gesamtzahl der erfassten Tage pro Teilnehmerin/Teilnehmer.

**Ergebnisse:** Dreiundsechzig Teilnehmerinnen und Teilnehmer (im Alter von 47,8±10,5 Jahren, 86% Frauen) wurden rekrutiert und begannen mit der Intervention unmittelbar nach der Eingangsuntersuchung. Zwei Personen schieden aus, alle anderen schlossen die Studie ab. Die Vereinbarkeit von TRE mit beruflichen Aktivitäten bewerteten 78% der Teilnehmer als gut, 3% als weder gut noch schlecht, während 18% von einigen Schwierigkeiten berichteten. Im Durchschnitt wurde das Fastenziel an 72,2±18,9% der erfassten Tage erreicht. Nach drei Monaten TRE zeigten die Teilnehmerinnen und Teilnehmer eine moderate Reduktion des Gewichts (–1,3±2,3 kg, p≤0,001) und des Taillenumfangs (–1,7±3,2 cm, p≤0,001). Die HRQoL stieg signifikant um 5,8±12,4 (p=0,008) Punkte zwischen Ausgangswert und Nachbeobachtung.

**Schlussfolgerung:** TRE ist gut durchführbar und auch bei berufstätigen Personen gut akzeptiert und kann die HRQoL verbessern. TRE kann dazu beitragen, Adipositas und abdominale Adipositas bei erwachsenen Berufstätigen zu reduzieren und damit lebensstilabhängige Krankheiten zu verhindern, aber die Teilnehmerinnen und Teilnehmer brauchen mehr Unterstützung, um die Wirkung zu verbessern.

## Introduction

The development of lifestyle-dependent diseases such as type II diabetes, cardiovascular disease, various cancers and chronic lung diseases is often correlated with overweight and obesity [1], [2], [3], [4]. The prevalence of overweight, obesity and abdominal obesity in the population is high and increases with age [5]. The consistently rising prevalence of overweight and obesity worldwide [6] shows that there has not been a breakthrough in prevention or treatment yet. A lasting change in lifestyle offers the greatest chance of preventing weight gain or achieving weight reduction [7]. Such lifestyle changes, which include an increase in physical activity and a change in eating habits, show good results under study conditions, but are rarely continued by those affected in the long term [8]. Nevertheless, the interest in diets or special forms of nutrition among the population remains unbroken. A relatively new trend is so-called intermittent fasting. Here the calorie supply is regularly reduced to ±500 kcal on fasting days. This can be alternated every second day (1:1), but also on one (6:1) or two non-consecutive days (5:2) of a week, whilst for the remaining days normally or ad libitum eating is intended, whereby the calories saved can be compensated [9]. The so-called “fasting-mimicking diet” comprises five days of calorie-reduced eating in three months, whereby the admission of proteins is likewise strongly reduced in order to simulate an approximate complete food renouncement with a special combination of plant-based food [10]. What all concepts of intermittent fasting described here have in common, however, is a reduced calorie intake on certain days. Altogether, successful intervention studies are available for these fasting forms, which confirm their effectiveness [11]. However, a certain degree of self-control is required for a consistent maintenance, at least concerning weight loss [12].

The “time-restricted eating” (TRE) regimen, however, works without a compulsory reduction in calories. As a general rule, people eat in a daily 8-hour time window, followed by a 16-hour fasting phase (“16/8”). This form of fasting, which has already been successfully tested in animal experiments, has a positive effect both on weight and on various metabolic parameters, and may even alleviate the consequences of a Western diet [13]. To date, there are only few studies available in humans, but these also show positive effects on the weight and metabolic parameters [9], [14]. For instance, a recent study reported that 10 hours of TRE improved weight, visceral fat and cholesterol in a group of 19 patients with metabolic syndrome [15]. In another pilot study with abdominally obese patients in a general practitioner’s office, we found that a daily restriction to 8–9 hours of eating improved weight, waist circumference and hemoglobin A1c (HbA1c) [16]. Furthermore, an earlier start of the eating phase at 8 am [17] seems to be more favorable than a later one at 12 pm. The latter did not even show a difference in weight loss compared to the control condition of three meals per day [18].

The study presented here investigates first and foremost the feasibility and adherence to an 8–9-hour limited period of food intake in everyday working life for healthy adult employees of Ulm University and University Hospital Ulm. A further focus is on side effects, participants’ attitudes, metabolic parameters and health-related quality of life.

## Materials and methods

This study was intended to provide information for a second pilot study with patients in a family practice and, together with the results of the latter, to deliver data for the design of a further randomized controlled trial. Hence, this TRE study was conducted in a pre-post design without a control group due to the pilot character of the study. The data collected were pseudonymized using a code to be created individually by the participant. The intervention was developed by a public health specialist and took place over a period of three months from September to December 2018. Participants were asked to limit their food intake to 8–9 hours per day to achieve an extension of the nightly fasting duration to 15–16 hours. There are no set guidelines for the time window in TRE studies, and we opted for the 8–9 hours to allow participants some flexibility. The primary target was the percentage of successful days with the planned restriction of the time of food intake in the total study period, considering a minimum of 70% of all recorded as sufficient. Secondary targets were mean differences between pre- and post-values in anthropometry, laboratory parameters, health-related quality of life (HRQoL) and sleep duration and quality.

Written informed consent was provided by all participants included in the study. The ethics commission of Ulm University approved the study (May 2018, application no. 153/18). The study was registered with the German Register of Clinical Trials in 2018 (DRKS-ID: DRKS00015057).

### Recruitment

Employees of the university and the hospital were informed about the study via flyers and in a lecture on TRE in the context of the operational health management of Ulm University in July 2018. Interested volunteers registered at the study center in the Institute of General Practice. Participation was limited to adult employees without known pre-existing metabolic conditions. There were no restrictions concerning weight or waist circumference. The number of participants was raised from 50 to 63 due to the great interest and the achievement of the recruitment target after only two weeks, and also in order to increase the proportion of male participants, which was initially very low.

### Data assessment

The initial examinations started in September 2018 and took place over a period of three weeks. In a personal consultation with a member of the study team, consisting of a medical doctor and a public health specialist, the participants were informed in detail about the content of the study and the implementation of the intervention. They received a brochure specifically developed for this purpose, with a detailed description of the fasting procedure and further evidence-based information on background and aim. They were asked to start TRE immediately after the initial examinations. If possible, the appointment for the follow-up examination was arranged exactly three months later.

The pre and post anthropometric measurements were carried out according to a standardized protocol. The participants’ height was measured to the nearest 0.1 cm (Stadiometer, Seca^®^, Germany), and body weight to the nearest 0.1 kg using calibrated and balanced portable digital scales (Seca^®^, Germany). Waist circumference (WC) was measured in the middle, between the iliac crest and the lower ribcage, using a flexible metal tape (Lufkin Industries Inc., Texas, USA). The Body Mass Index (BMI) was calculated (body weight/height in meters squared) and classified into normal weight (<25), overweight (≥25), and obese (≥30). The Waist-to-Height Ratio (WHtR) was calculated (WC/body height) and categorized as abdominal obesity from a threshold of 0.5. A blood sample was drawn to determine blood lipids (low-density lipoprotein (LDL), high-density lipoprotein (HDL), total cholesterol, triglycerides). Blood analyses were carried out at the Department of Clinical Chemistry at University Hospital Ulm, which is accredited according to DIN EN ISO 15189.

During the three-month intervention period, the participants documented the time of their first and their last meal as well as the duration of sleep in a pre-printed diary. The quality of sleep was assessed in the same diary with a visual analogue scale.

In December 2018, the follow-up examinations were carried out according to the same principle as the initial examinations, with questionnaires, anthropometry and blood sampling.

The participants completed a short questionnaire in order to collect information on their health behavior and lifestyle. Questions were partly based on questionnaires from previous studies and surveys [19], [20] or, with regard to specific variables, self-developed. At follow-up, the questionnaire was extended with questions on the feasibility, perceived health effects and side effects, and experience with and attitude towards TRE. Side effects as an important aspect of any intervention in humans were assessed in several questions. The first aimed at the occurrence of hunger outside the eating phase (never, less than once a week, once a week, several times a week, daily). The second asked about attacks of ravenous hunger, dizziness, nausea, and other side effects that could be reported in text form. A multi-selection was possible. The third question recorded the frequency of the side effects from the beginning of the implementation through less than once a week, once a week, several times a week to daily. The fourth question related to whether the side effects mentioned in questions one and two had improved in the course of the treatment. The possible answers were “yes”, “no”, and “I have not experienced any side effects”.

In addition to information on lifestyle and health behavior, the pre-questionnaire collected general data on occupational activity. The post-questionnaire then referred to possible changes in lifestyle and health in the past three months, perceived health effects and side effects, as well as to questions about the implementation of the intervention such as feasibility, and experience with and attitude towards TRE. HRQoL was assessed at baseline and follow-up by the Visual Analog Scale (VAS) taken from the EuroQol 5-Dimension (EQ-5D) questionnaire [21].

### Statistical analysis

The basic characteristics were explored descriptively for men, women and the whole group. Group differences between men and women were examined according to scale level and distribution with Fisher’s exact test for categorical data, and with Mann-Whitney U test, t-test or Welch’s t-test (for variance heterogeneity) for continuous data.

For the primary target adherence and feasibility, a descriptive evaluation with mean value, standard deviation, and range or frequency measures was calculated.

For the secondary targets, the mean difference between pre- and post-values was considered. The inference statistics were calculated according to the distribution and the scale level with a t-test for connected data or a Wilcoxon rank sum test.

Correlations between continuous variables were tested with the Pearson product-moment correlation coefficient.

The significance level for two-sided tests was set at α=0.05. All statistical analyses were carried out using the statistical software packages IBM SPSS Statistics for Windows, Version 25.0 (IBM Corp., Armonk, NY, USA).

## Results

Of the 63 participants included, two discontinued the study during its course due to illness and occupational stress; their follow-up data are missing. Two other participants were unable to maintain the restricted 8–9 hours of food intake due to incompatibility with family eating habits and illness, but continued the diary and participated in the final examination.

The baseline characteristics of the participants are shown in Table 1 (Tab. 1).

### Follow-up 

After three months of TRE, participants showed moderate reductions in weight (–1.3±2.3 kg) and WC (–1.7±3.22 cm). The reductions in weight were weakly negatively correlated with both the percentage of reaching the fasting goal (r=–.295, n=61, p=.021) and the average duration of fasting (r=–.306, n=61, p=.017). There was a slight increase in total cholesterol (0.34±0.51 mmol/l) which showed no significant correlation with the percentage of reaching the fasting goal or the average duration of fasting. HRQoL increased significantly by 5.8±12.4 points between baseline and follow-up. This increase correlated neither with fasting intensity nor with reductions in weight or WC. More details are given in Table 2 (Tab. 2).

### Primary outcome and diaries

**Figure 1:**
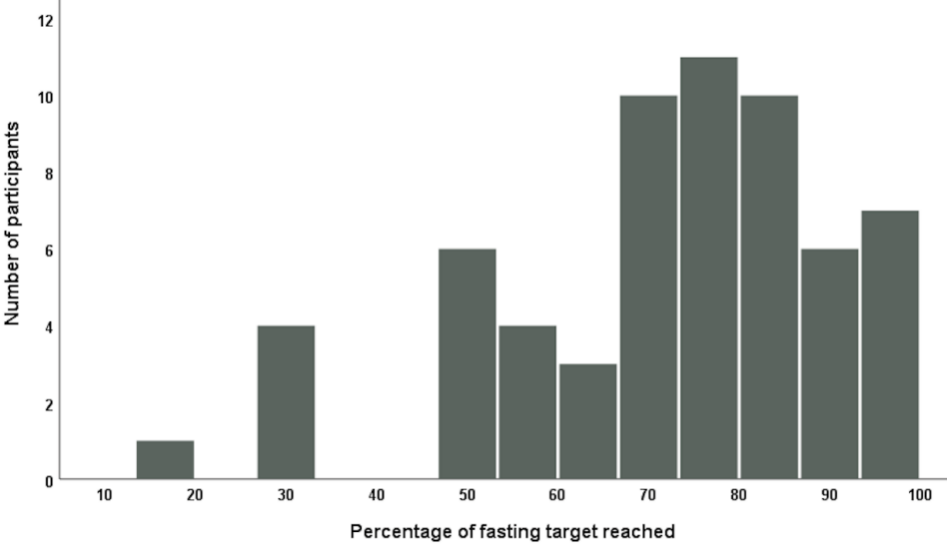
Percentage of days with attainment of the fasting target in the total number of documented days

On average, participants were able to reach the fasting goal of 15–16 hours in 72.2±18.9% of all recorded days (Figure 1 (Fig. 1)). There was considerable heterogeneity between participants regarding the individual percentage of reaching the fasting goal from 16.9% up to 97.7%.

**Figure 2:**
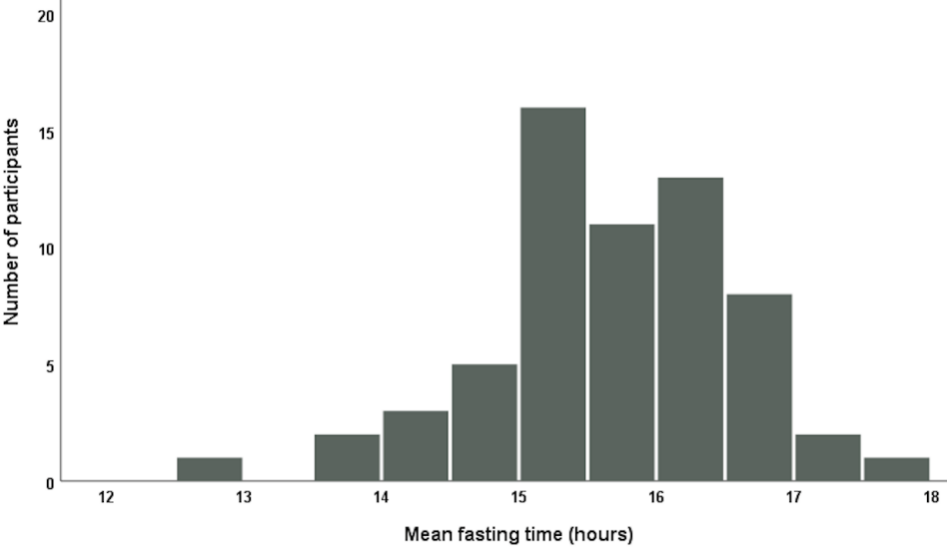
Mean fasting time of participants during the study course

Furthermore, the average fasting time of participants is illustrated in Figure 2 (Fig. 2). For all outcome variables, no significant differences between weight groups (normal weight, overweight/obese, abdominally obese) were detected. Weight loss differed between normal weight (–1.1±2.1 kg) and overweight/obese (–1.6±2.6 kg) participants, but the difference was not statistically significant. The same applies to the comparison of weight loss between participants without abdominal obesity (–0.9±2.2 kg) and those who were abdominally obese (–1.6±2.4 kg). Overweight participants lost –1.4±2.7 kg or 1.8%, while obese participants lost –1.9±2.6 kg weight, which is 1.9% of their initial weight.

Information from the diaries regarding daily eating and fasting time, sleep duration and sleep quality is depicted in Table 3 (Tab. 3).

### Side effects

Feeling hungry several times a week or on a daily basis was stated by 37% of participants. Never or less than once a week was reported by 44%, and once a week by 19%. Cravings, dizziness, nausea, or other side effects were reported by 29%, 24%, 15%, and 22%, respectively (multiple answers possible). Other side effects included circulatory problems, weakness, stomach pain, and headaches. 31% of side effects were only at the beginning of TRE, 49% occurred less than once a week or once a week, and 20% several times a week or daily. 45% reported that the side effects had improved during the course of the study, 28% claimed to have experienced no side effects.

### Participants’ assessment

The majority of the participants (78%) were able to combine TRE well with their professional activities, 3% considered it neither good nor difficult, and 18% reported encountering some difficulties. One participant dropped out because of occupational stress. More than half of the participants (53%) found it easy to stick to the restricted eating time, 25% said that it was difficult for them, and 22% rated it neither easy nor difficult. 60% rated TRE as positive for their health, 35% neither positive nor negative, and 5% as rather negative. 41% said they wanted to continue TRE, 55% were undecided, and 3% did not want to continue. Most participants (74%) would recommend TRE to others, 24% were undecided, and 2% did not want to recommend it. More than half (51%) would welcome it if TRE was offered preventively or therapeutically by their family doctor as an accompanied measure, 34% were undecided, and 15% were not interested. Finally, 34% rated their diet during TRE as better than, 46% as the same as, and 20% as worse than before. The proportion of participants who claimed to care or strongly care for their health increased from 58% at baseline to 72% at follow-up.

## Discussion

The primary target to investigate the adherence and feasibility of TRE in an adult working population showed good results in both areas. Participants reached the fasting goal in 72% of recorded days, and 78% reported good compatibility with their professional activity. This compatibility applies here to employees of a university, which are, however, composed of many different occupational groups with different tasks. Thus, TRE may have the potential to be conducted in a considerable proportion of the adult working population. Nonetheless, other researchers report that participants fear problems in adherence due to work schedules, family commitments and social events [22].

### Possibilities for improvement of TRE studies

With an average loss of –1.3±2.3 kg in weight and –1.7±3.2 cm in WC, mean differences were only small between baseline and follow-up data. However, there was considerable heterogeneity between participants, and weight change ranged from –8.9 kg to +3.2 kg, while changes in WC were between –13.3 cm and +4.1 cm. Changes in blood lipids showed more homogeneity, but did not deliver the expected improvements, apart from individual cases. Overall, these results are unsatisfactory with respect to the positive results from trials with rodents [13]. In addition to the fact that humans are not kept in cages and the feed supply cannot be controlled in terms of time and quantity, the absence of any dietary requirements or instructions is assumed to be another important reason for the moderate success. As a consequence, some participants may have eaten even more than usual because of fear of hunger in the fasting phase. In future studies, this problem has to be addressed explicitly. Participants should be given more instructions in terms of nutrition, and they need to be looked after more closely. These improvements may help participants who are not much aware of healthy eating or possibilities to avoid or minimize hunger with an appropriate selection of macronutrients before fasting. In our second pilot study with abdominally obese patients in a general practitioner’s office, some improvements were made, such as offering advice on how to avoid hunger during the fasting phase, and a telephone consultation was conducted after two to three weeks to identify and solve problems with TRE. The participants in this second study lost –1.7±2.5 kg weight and –5.3±3.1 cm WC [16].

### TRE and health-related quality of life

Independent of changes in body composition, there was a statistically and clinically significant increase in health-related quality of life. Although patient-reported outcomes are constantly gaining more interest and acknowledgment, this is to our knowledge one of the first measurements of HRQoL in intermittently fasting adults [23]. This result is especially important because it shows an increase in HRQoL independent of weight loss. Based on the complexity of HRQoL [24], it can be assumed that TRE may have positive physical and psychological effects, which are beyond the scope of this study to specify in detail.

### TRE, eating period, and weight loss

There are only few studies with small numbers of participants examining TRE in humans. The initial one by Gill and Panda used a mobile app and observed erratic eating patterns highly variable from day to day, with more than half of the 47 participating adults eating for 15 hours or more each day [25]. Furthermore, they report a bias toward eating late and consuming >35% of calories after 6 pm. Eight overweight individuals who reduced their daily eating time from >14 hours to 10–11 hours for 16 weeks subsequently reduced their body weight by 3.27 kg (95% CI: 0.908–5.624 kg) [25]. Our participants reported an average eating window of 12.4±1.8 hours before starting TRE. Eighteen overweight participants in our study reduced their weight by 1.38 kg (95% CI: 0.039–2.717 kg) in 12 weeks. The difference may be due to longer duration and regular individualized feedback in the study by Gill and Panda [25]. Additionally, as indicated by moderate but significant changes in WC and WHtR in our study, TRE may help to lose abdominal fat, an important fact for overall health, since abdominal obesity is associated with virtually all kinds of non-communicable diseases, and successful interventions are rare [26]. Gabel et al. report the effects of a pilot study of 8-hour TRE on body weight and metabolic disease risk factors in 23 obese adults [27]. Participants were allowed ad libitum eating between 10 am and 6 pm, and water fasting from 6 pm to 10 am for 12 weeks. Gabel et al. compared weight loss and other outcomes to a matched historical control group. Except for moderate changes in body weight (–2.6%), energy intake and systolic blood pressure, all other variables under consideration (LDL, HDL, triglycerides, fasting glucose, fasting insulin, homeostasis model assessment – insulin resistance (HOMA-IR), homocysteine) showed no significant differences to controls [27]. Compared to these results of Gabel et al. [27], the 11 obese participants in our study lost –1.9% body weight, confirming the moderate effects of a TRE regimen with ad libitum eating in obese participants.

### TRE, muscles, and exercise training

Gasmi et al. [28] investigated the influence of 12 weeks TRE on muscle performance and immune responses in 20- and 50-year-old men in groups of 10 persons each. They report that their 12 hours feeding – 12 hours fasting protocol decreases hematocrit, total white blood cells, lymphocytes, and neutrophils, but did not affect muscle performance [28]. Two other studies independently report results of randomized trials investigating TRE in young males performing resistance training. Moro et al. found a decrease in fat mass in the TRE group while fat-free mass and maximal strength were maintained [29]. Tinsley et al. report no changes in total body composition after the eight-week study period in the TRE group despite a reduced energy intake [30]. These findings confirm the results by Gasmi et al. [28] that TRE does not restrict the practice of exercise training. This is of course very important since fasting has always been associated with, and criticized for, muscle loss. Though not scientifically approved, TRE has, as “Leangains”, a large group of fans among the fitness scene, power athletes and bodybuilders, with the important message that fasting does not compulsorily mean a loss of energy and thus of muscle performance [31].

### TRE and time of eating

In addition to the duration of the period of food intake, the beginning of the eating time is also important, whereby an earlier start seems advantageous. Starting TRE as early as at 8 am improved 24-hour glucose levels, altered lipid metabolism and circadian clock gene expression [17]. Participants in our study started on average at 10:25 am with small improvements in anthropometric measures. Another study showed no significant difference in weight loss between an ad libitum eating period from 12 pm to 8 pm and a structured meal program [18]. Finally, Sutton et al. report a controlled feeding study with early TRE (eTRE) and the improvement of insulin sensitivity, blood pressure and oxidative stress, without weight loss, in pre-diabetic men [32]. Early TRE means that the eating window opens early in the morning. In this case, participants started to eat at 8 am and had their last meal before 2 pm. The underlying rationale is to eat in accordance with the circadian rhythms in metabolism. The control group had an identical meal plan, except for the timing, which started at 8 am and ended at 8 pm. The authors wanted to know whether their eTRE schema produces health benefits even without losing weight. After five weeks of controlled feeding, insulin sensitivity and β-cell function increased, while postprandial insulin, blood pressure, oxidative stress and appetite in the evening were reduced in the eTRE group [32]. Participants in our study had their first meal on average at 10:25 am and their last meal at 6:46 pm, and supposedly many of them skipped breakfast. There is evidence from several studies in humans and animals that eating at the time of the highest responsiveness of the endocrine system during the active phase of the day in accordance with the circadian rhythm optimizes the body’s food processing capacity [33]. Based on evidence mainly from animal studies, Patterson et al. propose a potential mode of action of intermittent fasting (IF), respectively TRE: The association of IF with lifestyle (diet, sleep and activity), the circadian central and peripheral clocks, and the diversity and activity of the intestinal microbiota may result in a metabolic regulation and subsequent reductions in obesity and other lifestyle-dependent diseases [34].

### TRE, advantages, and disadvantages

TRE offers several advantages over other forms of dietary interventions to prevent or treat weight problems and associated disease or disease risks:

- Low-threshold approach (meaning that the implementation does not necessarily have to be medically supervised)
- No calorie counting
- No dietary restrictions
- Individually adaptable to the daily rhythm
- TRE may improve health even without weight loss [32]

These advantages may be partly counteracted by some pitfalls:

- Continuation of a possibly unhealthy food selection
- Risk of overcompensation due to increased eating during the eating phase

The advantages of TRE make this approach particularly interesting for public health interventions, as the low barriers and ease of implementation can have a positive impact on both entry and adherence. Nonetheless, more research is needed to clearly identify positive and negative impacts in order to weigh the benefits against the risks.

### Strengths and limitations

We had only two drop-outs due to understandable reasons, which we consider very few. Overall, the adherence was very good thanks to the motivated participants. In comparison to earlier studies with observational character conducted by the authors, the number of missing data was very small. Unfortunately, the visual analog scale for the HRQoL was printed on the last page of the questionnaire so that 11 participants simply overlooked it in the baseline assessment. This may have led to an under- or overestimation of the longitudinal change. A minor strength of this research is probably its larger sample size compared to previous studies. With respect to the primary target of the study, one of the strengths is the heterogeneity of the participants with regard to their different professional activities.

The most obvious limitation is the missing control group. This was mainly due to the pilot character of the study and the primary targets, for which a control group was not absolutely necessary. Results therefore have to be interpreted with caution. Another limitation lies in the partially self-developed questions on specific information regarding TRE. Furthermore, a selection bias is possible due to the self-selection of the participants. It cannot be excluded that especially those who are already interested in a healthy lifestyle participated. Unfortunately, males are underrepresented, possibly reflecting their less pronounced interest in health and the lack of willingness to participate in self-management programs for chronic diseases [35]. Since this study was not funded, some examinations that would have been useful could not be carried out for financial reasons.

## Conclusions

TRE is feasible and well accepted, even in regularly employed persons, and may improve the HRQoL independent of changes in bodyweight. Furthermore, TRE may help reduce obesity and abdominal obesity in adult working people, thereby preventing lifestyle-dependent diseases. However, volunteers need more guidance to increase effects.

Further well-designed studies are necessary to investigate the possible benefits as well as the side effects of TRE and to develop a structured implementation scheme to ensure adherence and success.

## Notes

### Data availability

The datasets generated and analyzed during the current study are not publicly available due to reasons of data protection, but are available from the responsible data manager, Tibor Kesztyüs, on reasonable request.

### Competing interests

The authors declare that they have no competing interests.

### Informed consent

Written informed consent was provided by all participants included in the study.

### Ethical statement

The ethics commission of Ulm University approved the study (May 2018, application no. 153/18). The study was registered with the German Register of Clinical Trials in 2018 (DRKS-ID: DRKS00015057).

### Acknowledgments

First and foremost, we would like to thank all our participants. We are very grateful to Dr. Nanette Erkelenz and Dr. Meike Traub from the occupational health management of Ulm University for their friendly support. We would also like to thank the Division of Sports and Rehabilitation Medicine at the University Hospital Ulm for their support. Finally, we thank Sinéad McLaughlin for her valuable language assistance.

## Figures and Tables

**Table 1:**
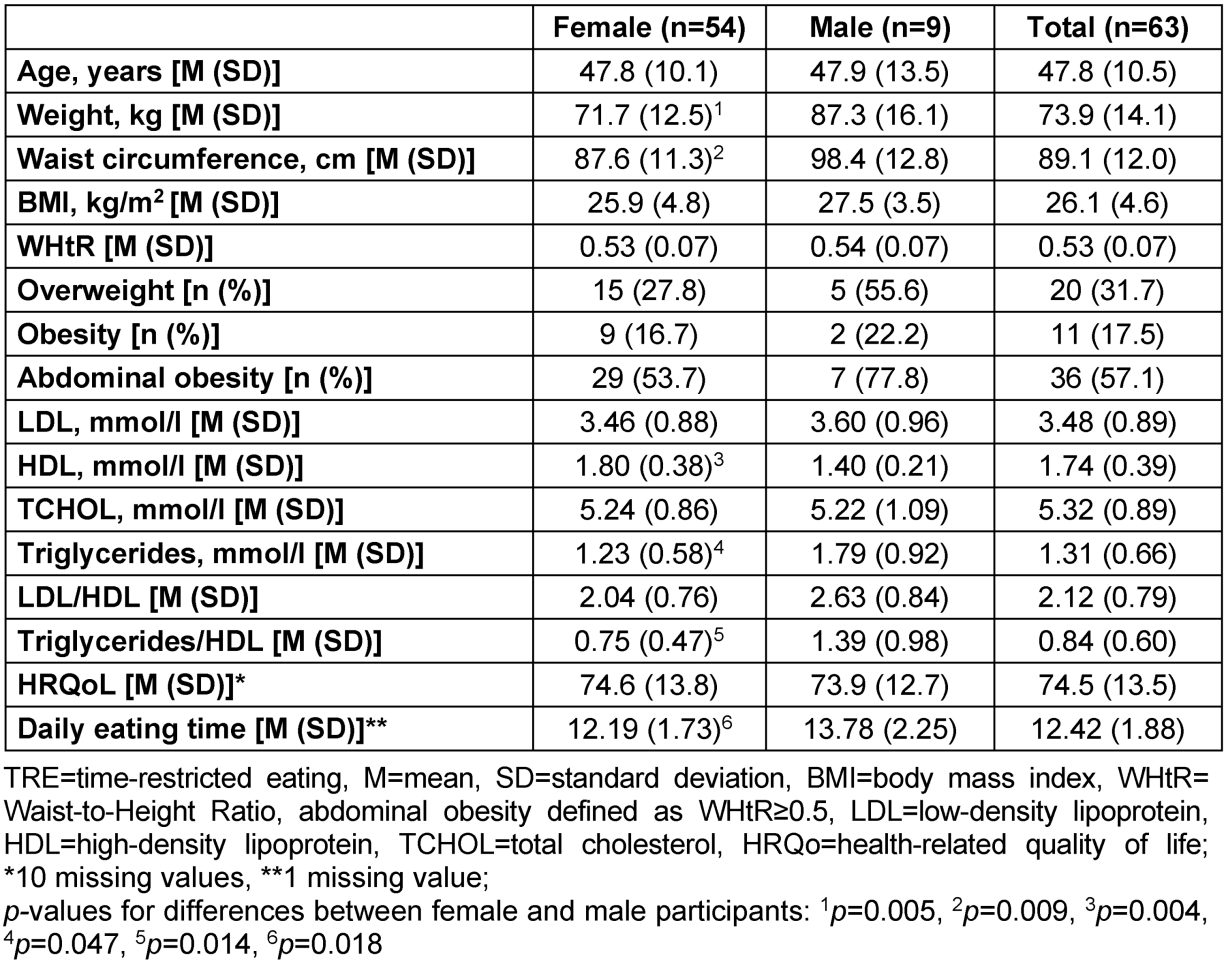
Baseline characteristics of participants in the TRE pilot study 2018

**Table 2:**
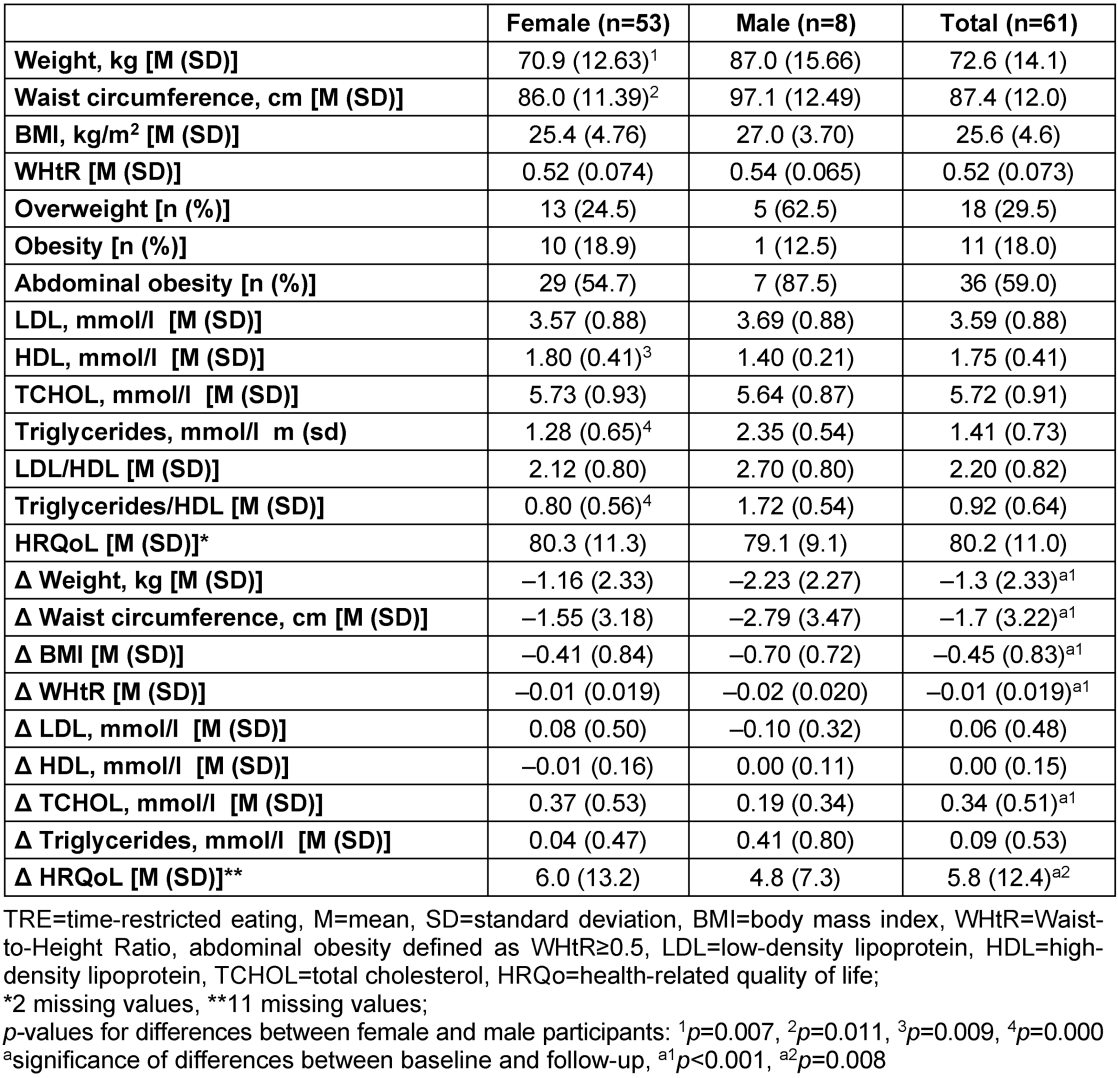
Follow-up results of participants in the TRE pilot study 2018

**Table 3:**
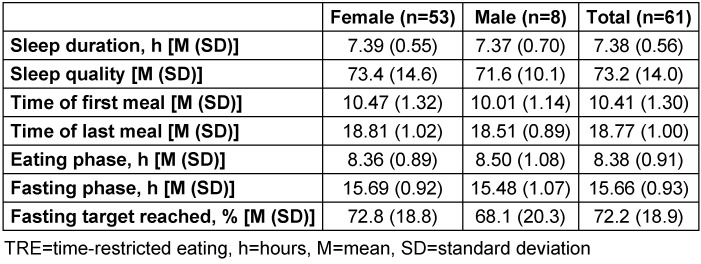
Diary of participants in the TRE pilot study 2018
